# MicroRNA-539-5p-Loaded PLGA Nanoparticles Grafted with iRGD as a Targeting Treatment for Choroidal Neovascularization

**DOI:** 10.3390/pharmaceutics14020243

**Published:** 2022-01-20

**Authors:** Jouri S. Alanazi, Fulwah Yahya Alqahtani, Fadilah Sfouq Aleanizy, Awwad A. Radwan, Ahmed Bari, Qamraa Hamad Alqahtani, Hosam Gharib Abdelhady, Ibrahim Alsarra

**Affiliations:** 1 Pharmaceutical Care Department, National Guard Health Affairs, Riyadh 11426, Saudi Arabia; ph.jouri@hotmail.com; 2 Department of Pharmaceutics, College of Pharmacy, King Saud University, Riyadh 11495, Saudi Arabia; faleanizy@ksu.edu.sa (F.S.A.); ialsarra@ksu.edu.sa (I.A.); 3 Kayyali Chair for Pharmaceutical Industry, Department of Pharmaceutics, College of Pharmacy, King Saud University, Riyadh 11451, Saudi Arabia; dhna_2001@hotmail.com; 4 Department of Pharmaceutical Chemistry, College of Pharmacy, King Saud University, Riyadh 11451, Saudi Arabia; abari@ksu.edu.sa; 5 Department of Pharmacology and Toxicology, College of Pharmacy, King Saud University, Riyadh 11495, Saudi Arabia; ghamad@ksu.edu.sa; 6 College of Osteopathic Medicine, Sam Houston State University, Conroe, TX 77304, USA; Hosam.abdelhady@shsu.edu

**Keywords:** choroidal neovascularization, internalizing RGD peptide, polylactic-co-glycolic acid nanoparticles, miR-539, angiogenesis

## Abstract

Choroidal neovascularization (CNV) is a major cause of visual impairment that results from excessive growth of blood vessels in the eye’s choroid. The limited clinical efficacy of the current therapy for this condition requires the emergence of new treatment modalities such as microRNA (miRNAs). A recent study identified microRNA-539-5p (miR-539) as an angiogenic suppressor in a CNV animal model; however, its therapeutic delivery is limited. Therefore, this study aims to formulate miR-539 in targeted nanoparticles (NPs) prepared from polylactic-co-glycolic acid (PLGA). The NPs were decorated with internalizing arginylglycylaspartic (RGD) peptide (iRGD), which specifically targets the alpha-v-beta-3 (αvβ3) integrin receptor that is overexpressed in blood vessels of ocular tissue in CNV patients. The ^1^H NMR spectra results revealed successful conjugation of iRGD peptide into PLGA NPs. The miR-539-PLGA.NPs and miR-539-iRGD-PLGA.NPs were prepared and showed a particle size of 300 ± 3 and 306.40 ± 4 nm, respectively. A reduction in human retinal microvascular endothelial cell (HRMEC) viability was shown 48 and 72 h post transfection with miR-539 incorporated in PLGA NPs and iRGD-PLGA.NPs. iRGD-functionalized PLGA NPs caused further significant reduction in cell viability when compared with plain ones, revealing an enhancement in the NP uptake with iRGD-grafted NPs. The current study showed that miR-539-PLGA.NPs and miR-539-iRGD-PLGA.NPs are promising approaches that reduced the viability of HRMECs, suggesting their therapeutic potential in the treatment of CNV.

## 1. Introduction

Choroidal neovascularization (CNV) is defined as growth of new blood vessels that originates from the choroid and extends into the subretinal pigment epithelium or subretinal space or both. CNV is a major cause of visual impairment due to leakage of immature blood vessels and subsequent fibrosis [1,2]. Current treatment of CNV involves vascular endothelial growth factor (VEGF) antagonists, such as bevacizumab and ranibizumab. Nevertheless, resistance to such monotherapy resulted in loss in visual acuity in a subset of treated patients [3,4]. Hence, new treatment options of CNV that act on different molecules are required to either substitute VEGF antagonists or to synergize their antiangiogenic effect.

The contribution of stromal cell-derived factor-1 (SDF-1), called CXCL12, in the development of CNV has been reported [5]. This effect is mediated through the interaction of SDF-1 with CXC chemokine receptor (CXCR) 4, leading to initiation of the recruitment of bone marrow-derived cells in CNV [5]. Inhibition of this interaction using CXCR4 antagonist or neutralizing antibodies was found to reduce the neovascularization in a laser-induced CNV model [6]. Moreover, recent studies have identified another chemokine receptor for SDF-1, designated as CXCR7, which plays a role in cell survival, adhesion, and mobility [6]. The expression of CXCR7 was found to be increased in CNV lesions and acted along with CXCR4 to regulate the tube formation of choroid–retinal endothelial (RF/6A) cells [7,8]. Importantly, a recent study has reported the overexpression of CXCR7 in retinal pigment epithelial/choroid/sclera tissues of laser-induced CNV rat [9]. Furthermore, an in vitro study revealed that blocking of CXCR7 activity by knocking down its gene or by using neutralizing antibodies suppressed SDF-1-enhanced survival and tube formation of human retinal microvascular endothelial cells (HRMECs) and reduced the size of CNV lesion in vivo [9].

MicroRNAs (miRNAs) are a class of small, noncoding RNAs that negatively regulate gene expression post-transcriptionally through binding to complementary sequences on the 3′UTR of the target mRNAs and typically silence genes [10,11]. Previous studies revealed the crucial function of miRNAs in the development and progression of wet age-related macular degeneration (AMD) [12,13,14]. A recent study has identified microRNA-539-5p (miR-539) as a regulator of CXCR7 [9]. Inhibition of the cell survival and tube formation was observed in HRMECs and choroid–retinal endothelial (RF/6A) cells transfected with miRNA-539 mimic. This suppressive effect of miR-539 was rescued by the overexpression of CXCR7 [9]. In addition, the intravitreal injection of miR-539 in vivo also revealed its antiangiogenic activities, in which involvement of ERK1/2 and AKT signaling downstream of CXCR7 was reported [9]. The results of this study suggested the therapeutic potential of miR-539 in CNV-associated diseases.

However, there are many limitations for the administration of miRNAs in vivo secondary to their susceptibility to RNase degradation. Therefore, nanoparticles (NPs), especially biodegradable ones, have been utilized for the gene delivery including miRNAs. Polymeric NPs provide a safe, less invasive tool that increases the drug concentration in the eye [15]. In addition, the use of NPs to treat ocular diseases allows targeted delivery, controlled release, and enhanced pharmacokinetics and finally improving the therapeutic efficacy of the drugs in the eye. In this regard, polylactic-co-glycolic acid (PLGA) is one of the most commonly used polymers. Moreover, the duration of nanoparticle delivery can be adjusted according to the PLGA degradation rate, which may span from months to years via fine-tuning the PLGA composition and molecular weight. The surface of PLGA NPs can be modified by a targeting moiety that allows the formulated NPs to target specific tissue. One of the examples of these targeting moieties are internalizing arginine–glycine–aspartic acid (iRGD) peptide and transferrin. RGD is a tripeptide containing arginine, glycine, and aspartate amino acid residues. The iRGD demonstrated binding affinity for both the αvβ3 integrin and neurophilin-1 (NRP-1) receptors [16]. It binds to αvβ3 integrin expressed on the surface of endothelium cells of blood vessels via its RGD motif. Then, the peptide is cleaved by proteolytic enzymes to CRGDK/R sequence (CendR motif) increasing the peptide affinity to the NRP-1, which is as a transmembrane protein, involved in mediating the peptide’s penetration into tissue [16]. Given the promising effect of miR-539 on a CNV animal model and the limitation of their in vivo administration, this study aims to formulate microRNA-539-5p in PLGA nanoparticles grafted with iRGD as a potential targeted therapy for CNV disease.

## 2. Materials and Methods

### 2.1. Materials

PLGA 50:50 (503H; intrinsic viscosity 0.32–30.44 dL/g) with molecular weight of 24,000–38,000 Da was purchased from Boehringer Ingelheim International GmbH (Ingelheim am Rhein, Germany). Dichloromethane (DCM), diethyl ether, polyvinyl alcohol (PVA), *N*-(3-Dimethylaminopropyl)-*N*′-ethylcarbodiimide hydrochloride (EDAC), and N-hydroxy succinimide (NHS) were obtained from Sigma-Aldrich Chemical Company (St. Louis, MO, USA). The miR-539 and negative control miRNA (miR-NC) were acquired from Dharmacon (GE Healthcare Dharmacon Inc. Company, Lafayette, CO, USA). iRGD (Ac-CCRGDKGPDC) with molecular weight of 1093 Da was obtained from ChinaPeptides (Shanghai, China). SDF-1 was purchased from Abcam (Cambridge, MA, USA). Furthermore, alamarBlue assay reagent was obtained from BioRad (Hercules, CA, USA). Human retinal microvascular endothelial cells (HRMECs) were obtained from the American Tissue Culture Company (Manassas, VA, USA). Endothelial cell medium kit (cat no. P60104) containing fetal bovine serum (FBS) and endothelial cell growth supplement (ECG) were obtained from Innoprot (Derio, Spain), while 0.25% trypsin with phenol red, antibiotic–antimycotic solution, and Quant-iT RiboGreen RNA Assay Kit were purchased from Invitrogen (Wilmington, DE, USA). DNA Gel Loading Dye and GeneRuler (50 bp) DNA Ladder were purchased from Thermo Scientific (Waltham, MA, USA).

### 2.2. Preparation of iRGD-Conjugated PLGA Polymer

The iRGD peptide was conjugated into the polymer using a carbodiimide coupling method as previously reported [17]. Briefly, 100 mg of PLGA was dissolved in DCM, and 4.8 mg of EDAC and 27 mg of NHS was added and stirred at room temperature for 24 h. Then, the solution was filtered, and activated PLGA was precipitated out in cold diethyl ether and kept drying under vacuum. The obtained activated polymer (80 mg) was dissolved in 1 mL DCM, and 5 mg of iRGD was added and stirred at room temperature for 24 h. The iRGD conjugated PLGA was isolated using cold diethyl ether, dried by lyophilization, and then washed with water and subjected to lyophilization for 48 h. The conjugation was assessed using NMR.

### 2.3. NMR

To verify the peptide conjugation, the ^1^H nuclear magnetic resonance (NMR) spectrum was utilized. The iRGD, PLGA, and iRGD-PLGA were dissolved in DMSO and detected by NMR (ASCEND Bruker 700 MHz; Switzerland Bruker Company, Billerica, MA, USA). The NMR spectrum was recorded in DMSO-d6 using standard ^1^H experiment with Bruker 700 MHz instrument equipped with cryoprobe. The total number of scans were 64, with an acquisition time of 3.99 s at 298 K temperature.

### 2.4. Preparation of PLGA Nanoparticles

PLGA NPs were prepared by the water–oil–water (W_1_/O/W_2_) double emulsion solvent evaporation method as described previously [18,19]. Briefly, 100 mg of PLGA 50:50 was dissolved in 1 mL DCM. Then, 20 nmole of miR-539 or miR-NC was dissolved in 200 or 400 μL of RNAase free water and mixed with DCM-containing solution. The mixture was emulsified by sonication at 24 W using ultrasonic processor (Branson ultrasonic processor, Thermo Fisher Scientific, USA) over an ice bath for 30, 60, 90 s to form the primary W/O emulsion. Then, 9 mL of 2% PVA was added to the primary emulsion and the mixture was sonicated at 24 W for 3 min in an ice bath to form the second W/O emulsion. To the double emulsion, 50 mL of 2% PVA was added and stirred for 3 h at room temperature using a magnetic stirrer (Heidolph, Germany). The NPs were retrieved by ultracentrifugation (20,000× *g* for 40 min at 4 °C). Retrieved NPs were washed twice with distilled water and finally lyophilized in the presence of 5% trehalose using Labconco Freeze Dryer (Kansas City, MO, USA). To prepare iRGD-PLGA-NPs, 90 mg of PLGA and 10 mg of iRGD functionalized PLGA were dissolved in 1 mL DCM and procedures were performed as described above. Empty NPs (blank-PLGA-NPs) were prepared exactly as described above without the addition of miRNA as control [18,19].

### 2.5. Determination of Particle Size and Zeta Potential

The zeta potential, polydispersity index (PDI), and particle size of the prepared nanoparticles were measured using Zetasizer Nano ZS90 (Malvern Instruments Ltd., Malvern, UK).

### 2.6. Quantification of Entrapment Efficiency (EE%)

After the centrifugation of the prepared nanoparticles, the amount of free miRNA in the supernatant collected was measured using Quant-iT RiboGreen RNA Assay Kit. The EE% of miRNA loaded in nanoparticles was determined as previously prescribed using Quant-iT RiboGreen RNA Assay Kit [20]. Briefly, the amount of unentrapped miR-539 in the nanoparticle wash solutions was determined using RNA binding dye RiboGreen. Fluorescence obtained from binding of RiboGreen dye to miRNA was measured using Microplate Reader Detection (Model Bio-Rad 550, Thermo Fisher Scientific, Wilmington, DE, USA) at an excitation wavelength of 480 nm and an emission wavelength of 520 nm. The amount of miRNA loaded into nanoparticles was determined by subtracting the total amount of miRNA recovered in the wash solutions (miRNA_w_) from the initial amount of miRNA (miRNA_i_) added. The control solution containing the same amount of microRNA added was used and subjected to the same method described above. EE% was calculated as follows:

$$\mathrm{miRNA}\;\mathrm{EE}\%=\left[\frac{{\mathrm{miRNA}}_{i}{\;-\;\mathrm{miRNA}}_{w}}{{\mathrm{miRNA}}_{i}}\right]\times 100$$

### 2.7. Characterization of Nanoparticles Morphology

The morphology of prepared nanoparticles was visualized and characterized using transmission electron microscope (JEM-1230EX; Tokyo, Japan) [21].

### 2.8. Stability Assessment of microRNA in PLGA Nanoparticles during Formulation

The stability of miRNA under various conditions during the emulsification process was investigated. At the beginning, the effect of sonication time on the integrity of miRNA was studied by sonication of solutions containing 0.26 mg/mL of miRNA at 24 W for 0, 30, 60, 90, and 120 s. These samples were further analyzed using 1% agarose gel electrophoresis. Then, the effect of PLGA on the stability of miRNA was examined by emulsification of 100 µL of microRNA (0.26 mg/mL) into 250 µL of DCM containing 25 mg of PLGA and subjected to sonication at 24 W for 0, 30, 60, 90, and 120 s to form the emulsion. This was followed by centrifugation at 20,000× *g* at 4 °C for 10 min, and then the supernatant was collected. To the supernatant, 200 µL of DCM and 500 µL of TE buffer were added. Then, the mixture was incubated at room temperature for 90 min to enable extraction of miRNA from DCM phase into aqueous phase. The aqueous phase was separated by centrifugation at 20,000× *g* at 4 °C for 20 min. Supernatant was collected, and residual DCM was removed by incubation of the solution at 37 °C for 5 min. The integrity of miRNA was analyzed by gel electrophoresis [22].

### 2.9. In Vitro Release Study of miRNA from Nanoparticles Formulation

A total of 50 mg of freeze-dried PLGA nanoparticles loaded with miRNA was suspended in 1 mL Tris-EDTA (TE) buffer in RNase free Eppendorf tubes and shaken in a water bath (10 rpm) at 37 °C. At predetermined time intervals, the tube was centrifuged at 20,000 rpm for 10 min. Then, the supernatant was collected for analysis and replaced with fresh buffer. Next, the amount of microRNA released was determined using a RiboGreen RNA Assay Kit (Wilmington, DE, USA) [18,19].

### 2.10. Cell Line Culturing

HRMECs were cultured in endothelial cell medium supplemented with endothelial cell growth supplement (ECGS), 5% fetal bovine serum (FBS), and 1% antibiotic–antimycotic. Cells were maintained at 37 °C in a humidified atmosphere with 5% CO_2_. The media were changed every other day until cells reached confluency. For seeding, cells were trypsinized and counted using a Scepter Cell Counter (Merck Millipore Co., Burlington, MA, USA).

### 2.11. In Vitro Transfection

Transfection was performed as previously described [9]. HRMECs cells were seeded onto 48-well tissue culture plates in culture media and left overnight at 37 °C. At 75% confluence, the cells were washed, and the media were switched to culture media without FBS and ECG and were incubated overnight. Then, miR-NC-PLGA.NPs, miR-539-PLGA.NPs, or miR-539-iRGD-PLGA.NPs were added to designated wells at 10, 25, and 50 pmole concentration and incubated with the cells for 5 h to mediate transfection. Following incubation, the media were removed, and the cells were washed once with 1X phosphate buffer saline (PBS), incubated with fresh culture media, and placed in the incubator for the following 24, 48, and 72 h. Next, the cells were prepared for further experiments.

### 2.12. Effect of MicroRNA-Loaded Nanoparticles on Cell Viability

Cell viability was detected using alamarBlue as described previously [23]. Briefly, nanoparticles containing miR-539 and miR-NC were added to endothelial cells at different doses for 24, 48, and 72 h. After experimental treatment, cell media were discarded and fresh media were added, and then alamarBlue was added to the cells at 10% of each well and cells incubated from 2 to 4 h at 37 °C, 5% CO_2_. Then, fluorescence was recorded at 550 excitations and 590 emission wavelengths using SpectraMax M5 (Molecular Devices, Silicon Valley, CA, USA). The viability of cells was expressed relative to cells treated with miR-NC viability (%) as follows:

$$\mathrm{Cell}\;\mathrm{Viability}\;(\%)=\frac{\mathrm{Fluorescence}\;\mathrm{of}\;\mathrm{cells}\;\mathrm{treated}\;\mathrm{with}\;\text{PLGA-miR-539}}{\mathrm{Fluorescence}\;\mathrm{of}\;\mathrm{cells}\;\mathrm{treated}\;\mathrm{with}\;\text{PLGA-NC}}\times \;100$$

After assessing optimal post-transfection interval, the transfected cells were further incubated with media containing SDF-1 at 100 ng/mL concentration for 24 h, then cell viability was determined using alamarBlue.

### 2.13. Statistical Analysis

The significance of the difference between groups was analyzed using one-way analysis of variance (ANOVA), taking *p* < 0.05 as the lowest acceptable threshold for significance. Results were expressed as averages ± SD.

## 3. Results

### 3.1. Characterization of iRGD Conjugation on PLGA Polymer

**Figure 1:**
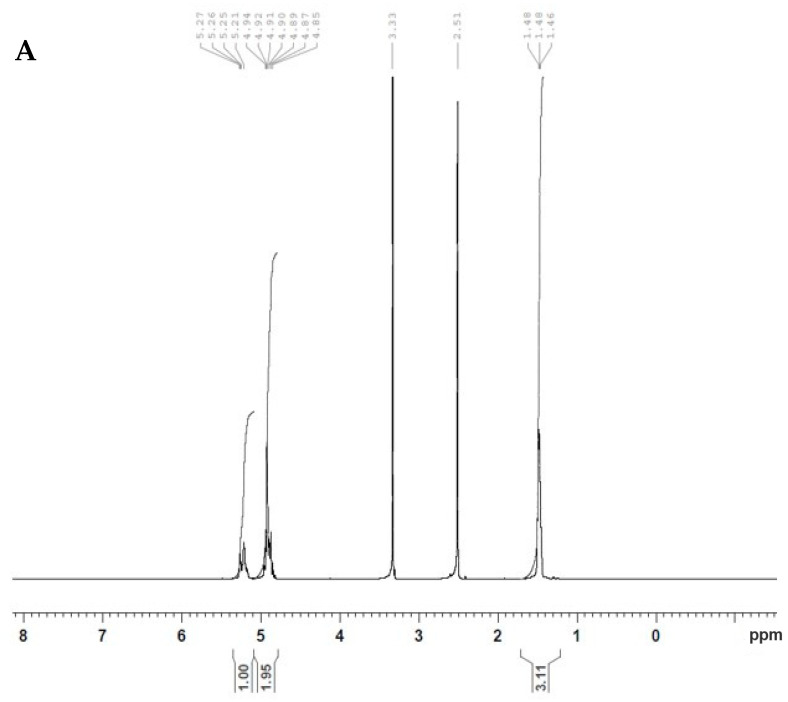

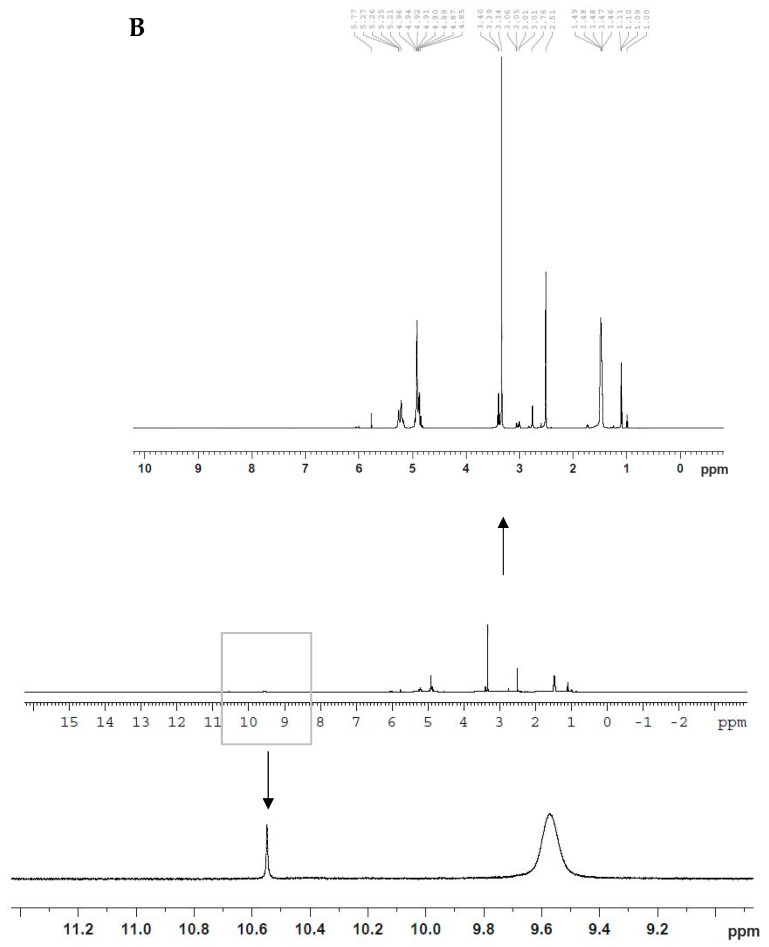
^1^H NMR spectra of PLGA (**A**) and PLGA-iRGD (**B**).

Conjugation of iRGD peptide to the surface of PLGA polymer was carried out as described previously [17] via a two-step process in which the polymer first was activated by sulfo NHS/EDAC, then NH_2_ of iRGD was attached to the activated polymer, forming a functionalized PLGA polymer. Furthermore, ^1^H NMR spectroscopy was used to characterize the conjugation between iRGD and PLGA. Figure 1A,B show the ^1^H NMR spectra of PLGA and PLGA-iRGD, respectively. In Figure 1A, various peaks are demonstrated at δ 1.47, 5.20, and 4.91 ppm, representing CH_3_ and CH from lactic acid and CH_2_ from glycolic acid, respectively. Figure 1B shows the ^1^H NMR spectrum of PLGA-iRGD conjugate. The peaks of iRGD appear small in addition to the signals of PLGA. The signals that appear at δ 1.1, 1.3, and 1.8 ppm characterize the central three methylene protons of arginine’s side chain. At δ 2.6, 2.76 ppm, signals appeared due to the -C-CH_2_-CO- of aspartic acid and C-CH_2_-N-arginine side chain protons and the peaks at δ 3.01–3.06 ppm characterize the cysteine CH_2_-S-, while the peaks at δ 3.4 ppm characterize the protons of amino acids α-carbons N-CH-CO and –CH_2_-N protons of proline amino acid. Moreover, two characteristic signals appeared at δ 9.52 and 10.53 ppm due to -CO-NH-amide protons of polyamides of iRGD itself and due to the PLGA-iRGD amide bond, respectively.

### 3.2. Preparation and Characterization of miR-539-Loaded PLGA NPs

The blank formula was successfully prepared and showed a nanosize of 290 nm with zeta potential of −6 mV (Table 1). When loading miRNA, the average particle sizes of miR-539 and NC-loaded PLGA NPs were 300 ± 3 and 300.20 ± 3 nm, respectively (Table 1). The diameter of iRGD-functionalized NPs increased by 6 nm (306.40 nm ± 4). All prepared NPs showed PDI ≤ 0.25, indicating uniform particle size distribution. All three formulations, miR-539-PLGA.NPs, miR-539-iRGD-PLGA.NPs, and miR-NC-PLGA.NPs, had negative zeta potential ranging from −16.4 ± 4.6 to −26.6 ± 5.16 mV (Table 1).

### 3.3. Gel Retardation Assay

**Figure 2:**
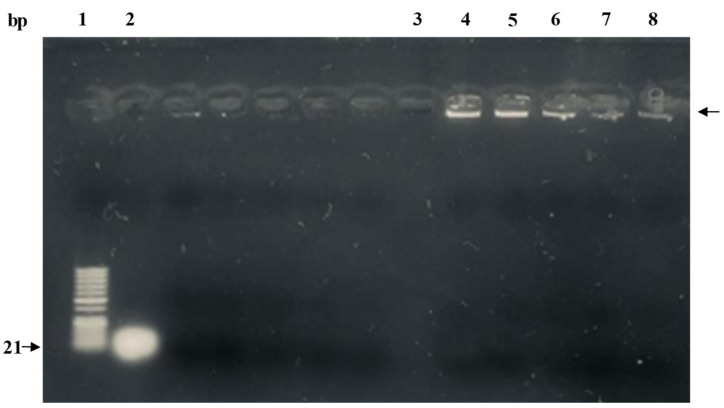
Gel retardation assay of miR-539-PLGA-loaded NPs. Lane 1: 50 bp ladder; lane 2: native miR-539 (positive control); lane 3: blank PLGA NPs; lanes 4–8: 0.2 mg of lyophilized miR-539-PLGA NPs were reconstituted in RNase free water and subsequently loaded in assigned lanes as demonstrated.

Gel retardation assay was performed to visualize the encapsulation of miR-539 in prepared PLGA NPs. As shown in Figure 2 (lanes 4–8), the miR-539-PLGA NPs showed greatly retarded migration in the gel when compared to naked miRNA (lane 2).

### 3.4. Stability Assessment of miRNA in PLGA Nanoparticles

**Figure 3:**
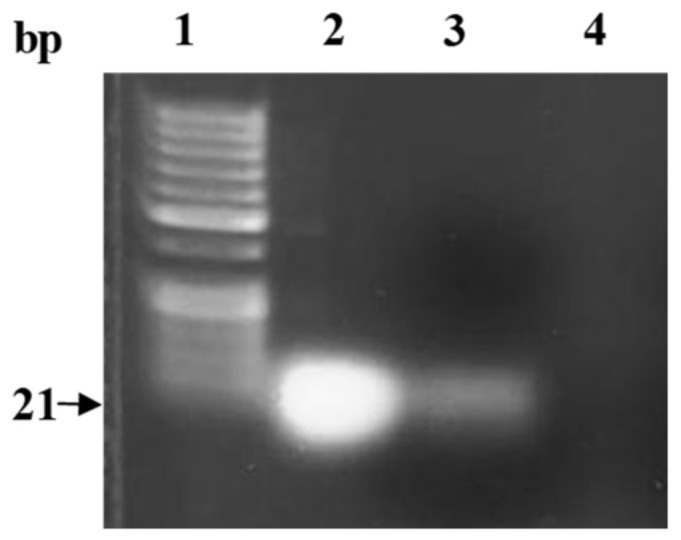
Gel electrophoresis of miR-539 extracted from the PLGA nanoparticles. Lane 1: 50 bp ladder; lane 2: native miR-539 (positive control); lane 3: extracted miRNA from 0.6 mg lyophilized PLGA NPs loaded with miR-539; lane 4: extraction from blank PLGA nanoparticles (negative control).

To ensure the integrity of miRNA in the final prepared formula, the miRNA was extracted from dissolved PLGA nanoparticles, and subsequently visualized by gel electrophoresis. As shown in Figure 3 (lane 3), the miR-539 was intact and preserved. This revealed that critical formulation steps including sonication and freeze-drying did not compromise the integrity of the miRNA.

### 3.5. Determination of EE%

As determined by RiboGreen assay, plain PLGA and RGD-grafted PLGA nanoparticles loaded with miR-539 showed 45% and 51.1% encapsulation efficiencies, respectively (Table 2). The EE% of PLGA NPs encapsulated with NC was 55.60% (Table 2). However, when the inner water phase increased from 200 to 400 µL, the EE% of miR-539-loaded PLGA NPs increased to 85% (Table 2), and 93% in NC-loaded PLGA nanoparticles.

### 3.6. Characterization of Nanoparticle Morphology

**Figure 4:**
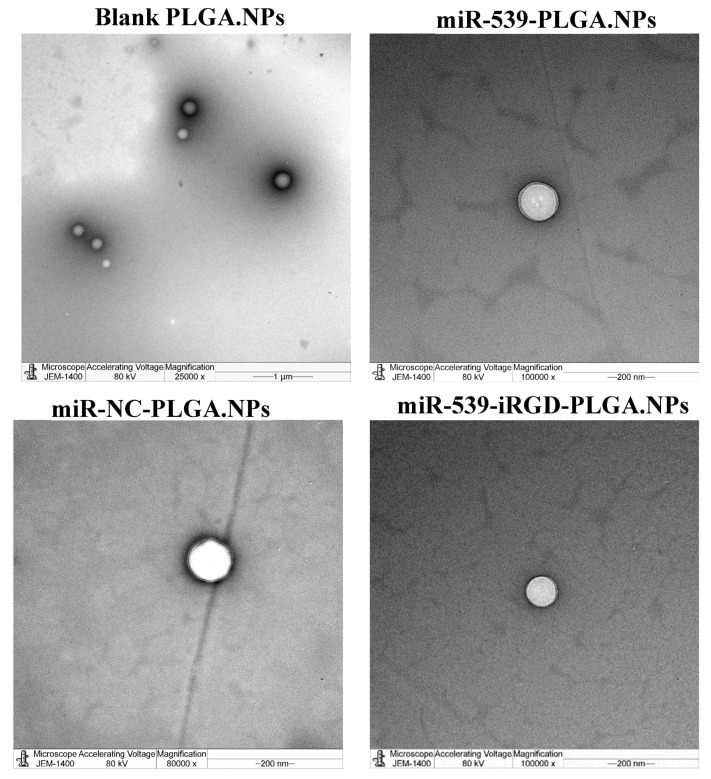
Transmission electron microscope of prepared PLGA NPs. Images of blank PLGA nanoparticles in scale range of 1 µm, loaded miR-539-PLGA nanoparticle in a scale of 200 nm, loaded miR-539-iRGD-PLGA nanoparticle in a scale of 200 nm, and loaded miR-NC-PLGA nanoparticle in a scale of 200 nm.

As presented in Figure 4, TEM images revealed spherical nanoparticles with a size ranging between 180 and 290 nm. These results are in compliance with particle size results measured by Zetasizer (Table 1).

### 3.7. In Vitro Release Study of miRNA from Nanoparticle Formulation

**Figure 5:**
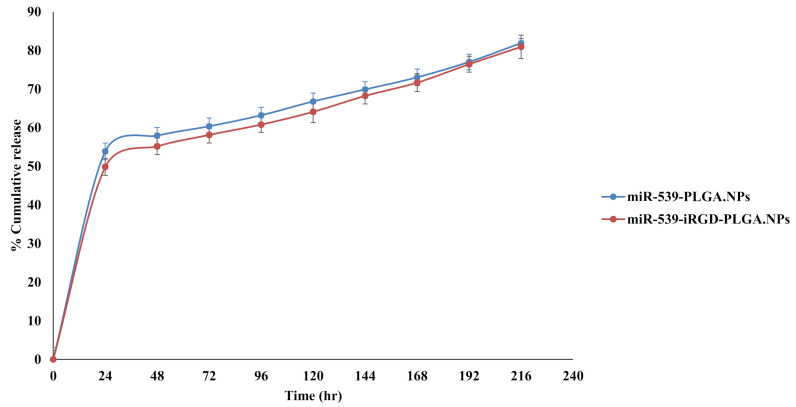
In vitro release of miR-539 from PLGA and iRGD-PLGA NPs. The release was evaluated in pH 7.4 using TE buffer, at 37 °C and 200 rpm. Points represent averages ± SD.

The in vitro miR-539 release profile form miR-539-PLGA and miR-539-iRGD-PLGA are shown in Figure 5. A burst release was observed during the first day, when 54% and 50% of loaded miR-539 was released from PLGA and iRGD-functionalized PLGA NPs, respectively (Figure 5). This was then followed by a slower and more sustained release over 9 days. Furthermore, the incorporation of iRGD into PLGA NPs did not change the release pattern of miR-539 from prepared NPs during the experimental period.

### 3.8. Effect of miR-539-PLGA Loaded Nanoparticles on Survival of HRMECs

**Figure 6:**
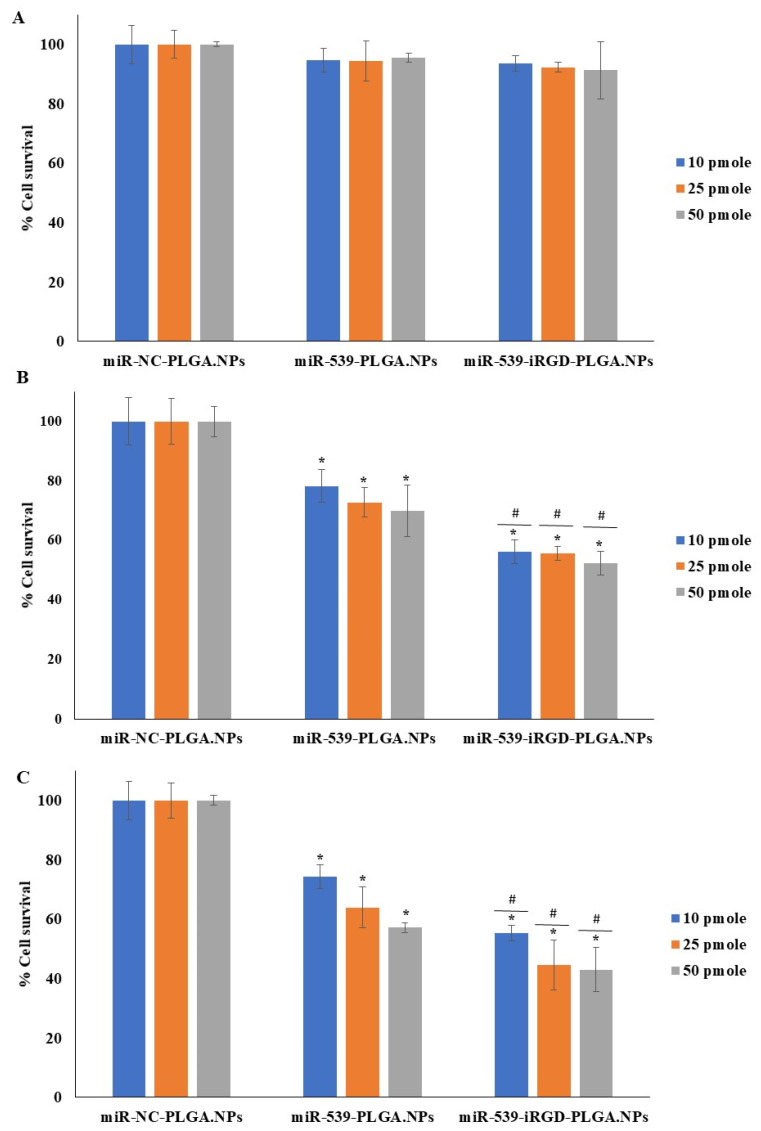
HRMEC viability after treatment with miR-539-loaded PLGA NPs and miR-539-iRGD-PLGA.NPs. Cells were treated with three different doses of the three NPs for 5 h, and then media were changed. Cell viability was assessed after 24 (**A**), 48 (**B**), and 72 (**C**) hours of treatment compared to cells treated with NC-loaded PLGA NPs. Data represent the mean ± SD, (*n* = 3). * and ^#^ *p* < 0.05 in ANOVA test relative to cells treated with NC-loaded PLGA NPs and relative to miR-539 plain NPs, respectively.

The cell viability of HRMECs was evaluated 24, 48, and 72 h post transfection with miR-NC-PLGA.NPs, miR-539-PLGA.NPs, or miR-539-iRGD-PLGA.NPs using alamarBlue, and the results are shown in Figure 6. After 24 h, there was no significant reduction in cell viability (Figure 6A). However, after 48 h, miR-539-PLGA.NPs reduced cell viability up to 78.3%, 73%, and 70% at 10, 25, and 50 pmole doses, respectively (Figure 6B). Further reduction in cell viability was observed in cells treated with iRGD-functionalized PLGA NPs to 56%, 55%, and 52% at 10, 25, and 50 pmole doses, respectively (Figure 6B). The viability of HRMECs dropped to 74%, 64%, and 57% after cell treatment with 10, 25, and 50 pmole of miR-539-PLGA.NPs for 72 h (Figure 6C), while in cells transfected with miR-539-iRGD-PLGA.NPs, viability decreased more, to 55.3%, 44.5%, and 43% at 10, 25, and 50 pmole doses, respectively (Figure 6C). For all subsequent experiments, a 10 pmole dose and 48 h post-transfection were chosen. This is because 10 pmole was the lowest concentration and, as observed, reduced cell viability significantly when compared to NC-loaded NPs.

**Figure 7:**
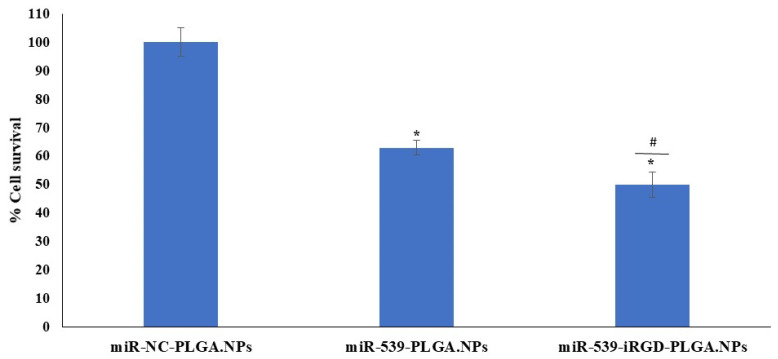
HRMEC viability after treatment with miR-539-loaded PLGA NPs and SDF-1 stimulation. Cells were treated with 10 pmole of NPs for 5 h, and then media were changed. Cell viability was assessed after 48 h of treatment compared to cells treated with NC-loaded PLGA NPs, and then media were replaced with media containing 100 ng/mL of SDF-1 for 24 h. Data represent the mean ± SD, (*n* = 3). * and ^#^ *p* < 0.05 in ANOVA test relative to cells treated with NC-loaded PLGA NPs and relative to miR-539 plain NPs, respectively.

Furthermore, the induction effect of SDF-1 on the angiogenesis of HRMECs transfected with miR-NC-PLGA.NPs, miR-539-PLGA.NPs, or miR-539-iRGD-PLGA.NPs was investigated. As shown in Figure 7, cell viability was not restored in cells transfected with PLGA-miR-539 and iRGD-PLGA-miR-539.

## 4. Discussion

Angiogenesis is the growth of new blood vessels from pre-existing ones. In pathological conditions, excessive and dysregulated angiogenesis leads to neovascularization, which is associated with many diseases, including CNV [24]. In this regard, CNV is the hallmark of wet age-related macular degeneration (wAMD) and usually results in vision loss [25]. The role of miRNAs in modulation of angiogenesis, particularly in CNV [26], has been established recently [27], in which miRNAs contribute in regulating cellular response to angiogenic stimuli. As reported by numerous researchers, many microRNAs showed the ability to inhibit CNV such as miR-539 [9]. This was indicated by their suppression of proliferative, migratory, and tubulogenic formation in microvascular endothelial cells transfected with these miRNA mimics [26]. Therefore, miRNA is considered as promising therapeutic molecules in ocular diseases. Nevertheless, the use of these promising therapeutic molecules is limited due to their poor stability in physiological conditions and their inability to cross cellular membrane. Therefore, several nanocarrier strategies were utilized to improve physico-chemical and biopharmaceutical properties of miRNA [27]. Among these strategies was the entrapment of miRNA into biodegradable PLGA nanoparticles, which are widely used as a gene delivery system [19,20,21]. This is due to their sustained release characteristic, biodegradability, biocompatibility, capacity to overcome gene degradation by lysosomes, and their ability to offer physical shields against RNase activity which results in maintaining the safe delivery of miRNA to its target [28]. Hence, the present study was designed to formulate PLGA and iRGD grafted PLGA NPs encapsulating miR-539 as potential therapy for CNV disease.

Considering targeting of the prepared NPs to the posterior segment of the eye, the surface modification of the PLGA using targeting moiety such as iRGD would improve binding to the eye tissue and subsequent efficient delivery of the gene as reported previously [19,29]. The functionalization process of PLGA performed in our study is based on a carbodiimide coupling strategy utilized extensively by other researchers [17,19,30]. NP functionalization could be performed either before or after NP formation; we considered conjugation of the iRGD to the PLGA before NPs construction to overcome leakage of miRNA when functionalization performed after NP formation. Then, the iRGD-PLGA NPs were formulated by combining 10% iRGD grafted PLGA and 90% plain PLGA. The NMR results revealed successful conjugation of iRGD into the surface of PLGA polymer as proved by ^1^H NMR spectroscopy results, which showed the formation of the amide bond between the peptide and the polymer.

The findings in this study revealed no significant change in the particle size after loading with miR-539 and miR-NC into PLGA, when compared with blank PLGA NPs. However, the size of iRGD-grafted PLGA NPs increased by 6 nm, attributed to iRGD conjugation. Such an increase in particle size of functionalized NPs is in line with the study findings of Singh et al. (2009), in which RGD-grafted PLGA-Flt23K NPs had a particle size of 382.2 ± 1.5 nm, which was larger than non-grafted PLGA-Flt23K NPs (375 ± 4.0 nm) [19]. A similar observation was made in the study by Luo et al. (2013), with a larger increase in particle size between RGD-conjugated (572.45 nm) and non-conjugated (522.91 nm) [30]. Consistent with previous reports [17,30], this research found that the presence of iRGD increased the zeta potential of miR-539-PLGA-NPs to –16 mV, while in non-grafted NPs, the zeta potential was −26 mV. This is because iRGD peptide contains positively charged amino acid that neutralizes the negative charge on the surface of the NPs, leading to an increase in zeta potential. Ensuring the stability and integrity of miRNA encapsulated in polymeric NPs is crucial in miRNA-based therapeutics. Therefore, the miR-539 was extracted from dissolved PLGA NPs and subjected to gel electrophoresis. As the results show, miR-539 appeared intact and preserved, revealing that the formulation process involving sonication and freeze drying did not compromise miR-539 integrity as reported in earlier studies using other miRNA and siRNA [22,31,32].

The encapsulation efficiency of miR-539 obtained was 45% and 51.1% in plain PLGA and iRGD-grafted PLGA nanoparticles, respectively. These percentages are slightly lower than those observed in the 2010 study by Zhang et al., [33], in which encapsulation efficiency of short hairpin RNA pDNA that targets hypoxia-inducible factor-1α (HIF-1α) into PLGA NPs was 60.2%. PLGA polymer is known to be hydrophobic and has a negative charge; thus, it cannot condense with negative or hydrophilic miRNA by electrostatic interaction. Therefore, the most feasible method to encapsulate genes including miRNA into PLGA NPs is the double emulsion solvent evaporation method. Such entrapment is challenging and requires optimization of the formulation process [27]. Notably, one of the important steps during formulation is the volume ratio of external aqueous phase to organic phase (*V_w_*/*V_o_*). In agreement with reports published previously [22,34], in this study, when the ratio of *V_w_*/*V_o_* increased to 0.4, the EE% of miR-539-loaded PLGA NPs increased to 85% and 93% for NC mimics. An explanation of the increase in EE% as the *V_w_*/*V_o_* ratio increased is that when the water phase volume increased, it would reduce the miRNA concentration gradient between the inner and outer water phase. Hence, this limits diffusion of miRNA from the inner to the outer water phase and subsequently increases the encapsulation efficiency [22]. Conversely, researchers often incorporated PLGA with polycations such as PEI [29,31,35], which can interact with RNAi by electrostatic interaction. Although this increases encapsulation efficiency, the reported cytotoxicity with these polycations remains a central concern [27]. Therefore, most studies [5,17,18,19,30], including our current study, avoid using polycations and consider the double emulsion solvent evaporation method to entrap genetic materials inside PLGA NPs.

As observed from our in vitro release results, iRGD functionalization of PLGA did not affect the release of miR-539 from formulated NPs and exhibited a comparable pattern to plain PLGA NPs, which is in accordance with previous studies [31,36]. Zhang et al. (2010) developed PLGA NPs encapsulating plasmid of pshHIF-1 [33]. When they evaluated the in vitro release of these NPs, they found that 77% of plasmid was released within 10 days, followed by constant sustained release for 4 weeks [33]. In our study, a proximity of 82% and 81.30% of miR-539 was released from PLGA and iRGD PLGA NPs, respectively, within 9 days. Our results were comparable with Zhang et al. (2010). The in vitro release findings of the current study further support the sustained pattern of PLGA, which is a common finding in PLGA NP systems [37].

In the current study, the capacity of formulated NPs to transfect HRMECS with anti-angiogenic miR-539 was evaluated. As demonstrated by cytotoxicity studies, there was no significant reduction in cell viability after 24 h, while after 48 and 72 h, cell viability was reduced in cells treated with miR-539-PLGA-NPs in a dose- and time-dependent manner. A further significant reduction was observed in cells treated with iRGD-functionalized PLGA NPs loaded with miR-539. Such a reduction in HRMECs was not restored in cells transfected with miR-539-PLGA.NPs or miR-539-iRGD-PLGA.NPs after their induction with SDF-1. Cumulatively, these findings indicated that iRGD NPs produced a greater reduction in cell viability when compared with plain NPs. This might be explained by the capacity of iRGD-grafted NPs to enter cells more efficiently than plain ones as demonstrated by earlier reports [31]. However, both miR-539-loaded PLGA and iRGD-PLGA NPs reduced cell viability. Therefore, although the uptake by the cells of functionalized NPs was large, both miR-539-loaded PLGA NPs share the same intracellular fate in which they mainly circumvent endo-lysosomal degradation. This allows for the sustained release of miR-539 from NPs as shown in the study by Devalliere et al. (2014), in which they found that both cRGD-targeted and non-targeted PLGA NPs are endocytosed through a clathrin-dependent mechanism [31]. Thus, the NPs will serve as a depot within an intracellular vesicular compartment that slowly releases siRNA into the cytosol, protecting it from degradation as reported by other researchers [31].

MiR-539 is a regulator for downstream events that regulates retinal endothelial cell proliferation, tube formation, and angiogenesis [9]. Given that, future studies should examine the effect of prepared NPs on the expression of effector proteins and tube formation in HRMECs. Further in vivo research using a laser-induced CNV rat model will support characterization of the anti-angiogenic effects of miR-539-loaded PLGA and iRGD-PLGA NPs and, subsequently, their potential clinical implications for treating CNV patients.

## 5. Conclusions

The current study showed that miR-539-PLGA and miR-539-iRGD-PLGA NPs are potential promising therapeutics for CNV, as they provide sustained release of miR-539 within transfected cells, allowing their biological action, involving reduction in SDF-1-induced survival of HRMECs, to persist for an extended period of time.

## Figures and Tables

**Table 1:** Physicochemical characteristics of miRNA-loaded PLGA nanoparticles. The particle size, PDI, and zeta potential of blank, miR-539 and NC loaded PLGA, and iRGD functionalized NPs were measured. Data represent average ± SD, (*n* = 3).

Formula	Particle Size (nm) ± SD	PDI ± SD	Zeta Potential (mV) ± SD
miR-539-PLGA.NPs	300 ± 3	0.08 ± 0.02	−26.6 ± 5.16
miR-539-iRGD-PLGA.NPs	306.40 ± 4	0.25 ± 0.02	−16.4 ± 4.6
miR-NC-PLGA.NPs	300.20 ± 1.5	0.14 ± 0.03	−19.2 ± 5.26
Blank.PLGA.NPs	290 ± 2.51	0.11 ± 0.04	−6 ± 0.2

**Table 2:** Effect of volume ratio of the inner water phase to the oil phase on the EE% of prepared PLGA NPs. The concentration of free miR-539 was calculated from the standard curve. Samples were studied in triplicate and data represent mean ± SD. Free (unbound) miR-539 was measured by recording the fluorescence of supernatant of prepared NPs. *V_w_* represents volume of the inner water phase, and *V_o_* represents volume of oil phase.

Formula	*V_w_* (µL)	*V_w_*/*V_o_*	EE% ± SD
miR-539-PLGA.NPs	200	0.20	47.20 ± 1.84
	400	0.40	85 ± 0.62
miR-539-iRGD-PLGA.NPs	200	0.20	51.10 ± 0.50
	400	0.40	85.1 ± 1.80
miR-NC-PLGA.NPs	200	0.20	55.60 ± 3.50
	400	0.40	93 ± 0.24
